# N_2_^+^-implantation-induced tailoring of structural, morphological, optical, and electrical characteristics of sputtered molybdenum thin films

**DOI:** 10.3762/bjnano.16.38

**Published:** 2025-04-01

**Authors:** Usha Rani, Kafi Devi, Divya Gupta, Sanjeev Aggarwal

**Affiliations:** 1 Ion Beam Centre, Department of Physics, Kurukshetra University, Kurukshetra-136119, India

**Keywords:** atomic force microscopy, grazing angle X-ray diffractometer, Keithley parametric analyzer, Mo thin films, RF sputtering, spectroscopic ellipsometry

## Abstract

Molybdenum (Mo) thin films have extensive applications in energy storage devices and photovoltaic solar cells because of their remarkable thermal stability, high melting point, and chemical inertness. In the present study, Mo thin films of different thicknesses (150, 200, 250, and 300 nm) have been deposited on Si(100) substrates via radio frequency sputtering in an argon atmosphere at room temperature. Some of these films have been implanted with 1 × 10^17^ N_2_^+^·cm^−2^ at 30 keV using a current density of 4 µA·cm^−2^. Surface morphology and structural, optical, and electrical properties of the as-deposited and implanted Mo thin films have been systematically investigated. The crystallinity of Mo thin films is enhanced with increasing thickness of the as-deposited films. This pattern persists with film thickness even after N_2_^+^ implantation. After implantation, crystallinity decreases relative to as-deposited films with the same nominal thickness. The AFM analysis reveals that RMS roughness increases with the thickness of Mo films. Optical studies using spectroscopic ellipsometry reveal a significant increase in absorbance and reflectance in as-deposited and N_2_^+^-implanted films. Electrical investigations show that the conductivity increases with film thickness in both as-deposited and implanted films. The conductivity decreases for the same nominal film thickness after implantation.

## Introduction

Molybdenum thin films have garnered significant attention in diverse technological applications owing to their outstanding characteristics. The high melting point and stability of molybdenum ensure that it remains structurally intact under the harsh operating conditions of solar cells [1–2]. This stability is essential for long-term reliability and performance. The low resistivity of Mo thin films makes them desirable for integrated circuits, where they contribute to the efficient flow of electrical current [3]. Furthermore, their optical properties make them well suited for a use as a protective coating in energy storage and electronic devices [4–5]. Mo films deposited on substrates under suitable conditions lead to improvements in functionality and address the needs of various cutting-edge industries [6–7].

For the deposition of Mo thin films, various techniques such as chemical vapor deposition, physical vapor deposition (RF sputtering and DC sputtering) [1,8–9], and electron beam evaporation [10] have been reported in the literature. RF sputtering is the predominant technique for thin film deposition because of its benefits regarding layer adhesion, uniformity, composition, and deposition rate compared to other methods [11]. In the deposition of molybdenum films, RF sputtering allows for precise control of film thickness, shape, and stoichiometry, making it a key method for preparing films with specific characteristics [12].

Numerous approaches exist to improve the performance of thin films, including ion implantation techniques that enable precise alteration of material characteristics. Ion implantation is one of the most attractive techniques because it introduces considerable changes in the surface morphology and composition of the films [13–14]. The uses of implanted Mo thin films cover a broad range of applications including microelectronics and optoelectronics. One major use area is in microelectromechanical systems, where these films are used in parts such as microsensors, actuators, and microfluidic devices [15].

In this regard, the effects of different ion beams on the characteristics of Mo thin films have been explored [13–21]. Ahmed et al. [13] investigated the effect of helium ion irradiation on the structural and electrical properties of Mo thin films. They noted that α-particles create defects that reduce charge carrier mobility, and the hardness increased from low to high ion fluence. Hoffman et al. [14] investigated the effects of argon ion bombardment on the characteristics of Mo thin films. Films with reduced porosity and larger grains demonstrated increased reflectance and decreased resistivity, consistent with electron mean free path analysis results. Navin et al. [15] investigated self-organized pattern generation in Mo thin films with a low-energy argon ion beam (1 keV) across different ion fluences (10^16^–10^18^ ions·cm^−2^). Thornton et al. [16] examined a transition from tensile to compressive stress in argon-ion-implanted Mo thin films as the sputtering gas pressure decreased. Sun et al. [17] also analyzed the properties of argon-ion-implanted Mo thin films deposited via ion beam sputtering, varying deposition parameters such as accelerating voltage, incidence angle, and chamber pressure. Films deposited at near-normal incidence exhibited compressive stress and a nearly linear increase with the accelerating voltage. At grazing incidence, the observed stress is either minimal or slightly tensile and is mostly unaffected by the accelerating voltage. Tripathi et al. [18] examined the temperature-dependent surface alterations in Mo films induced by He^+^ ion irradiation within the 773–1073 K range as a prospective substitute for tungsten in plasma-facing components of fusion devices. Klaver et al. [19] investigated the impact of irradiation with low-energy helium ions on the physical properties of molybdenum thin films. Ono et al. [20] studied the degradation of the optical characteristics of single- and polycrystalline Mo mirrors for plasma diagnostics when treated with low-energy He^+^ ion irradiation at ambient temperature and 400 °C. Takamura et al. [21] examined the effects of He plasma irradiation on Mo thin films. The temperature range for nanostructure growth was within a temperature range of 800 to 1050 K, under incident helium ion energies of 50 to 100 eV. Nitrogen gas offers a fascinating opportunity for ion implantation in Mo thin films because of its high reactivity [22]. The incorporation of nitrogen ions alters the characteristics of Mo thin films, potentially improving their performance in a wide range of applications [23–28]. Kim et al. [23] examined the impact of a 3 × 10^17^ N_2_^+^·cm^−2^ ion fluence on the structural characteristics, surface morphology, and thermal stability of Mo thin films. The internal stress of these films transitioned from strongly compressive to weakly tensile after annealing. Lee et al. [24] efficiently incorporated nitrogen ions into epitaxial Mo films, forming a buried superconducting layer. They deposited atomically flat epitaxial Mo(110) films on Al_2_O_3_(0001) substrates. Carreri et al. [25] investigated high-temperature (800–1200 °C) plasma-based nitrogen ion implantation on molybdenum thin films. They also examined the phase development and tribological alterations caused by ion implantation. Furthermore, Nakano et al. [26] investigated the deterioration of optical characteristics in polycrystalline Mo mirrors exposed to irradiation with helium or deuterium ions. With increasing fluence and energy of the ions, a greater extent of deterioration was observed in helium-irradiated specimens than in deuterium-irradiated specimens. Mändl et al. [27–28] examined the impact of nitrogen ion implantation on Mo, focusing on nitride phase formation and nitrogen diffusion behavior within a temperature range of 330 to 580 °C. They observed the formation of a new cubic Mo_2_N phase. In addition, they also examined the impact of high ion fluence and temperature on nitrogen implantation in molybdenum with supplementary heating within the temperature range of 500 to 750 °C.

It is well documented that ion beam implantation, including helium, nitrogen, and argon ions, is a versatile technique for tuning the properties of molybdenum thin films. Researchers examined the effects of ion fluence, implantation temperature, and ion energy on the characteristics of Mo thin films. Despite these advances, a noticeable gap exists in the systematic exploration of nitrogen implantation with variations in the thickness of Mo films. To address this gap, this study employs an extensive approach, implementing low-energy nitrogen ion implantation with a systematic variation in film thickness and investigating the effects of these factors on the characteristics of molybdenum thin films. This study promotes the development of next-generation Mo thin films with precisely tuned characteristics. The findings of this research contribute to the optimization and development of Mo thin films for various applications in photovoltaics, sensors, optoelectronics, and electronic devices.

In the present work, Mo thin films with varying thickness of 150, 200, 250, and 300 nm were deposited on Si(100) substrates using radio frequency (RF) sputtering in an argon environment at ambient temperature. Some films with different thicknesses were implanted with 1 × 10^17^ N_2_^+^·cm^−2^ at 30 keV using a current density of 4 µA·cm^−2^. This study illustrates the effects of nitrogen ion implantation and film thickness on the structural, optical, and electrical properties of thin molybdenum films.

## Experimental

In this study, molybdenum thin films of varying thickness were deposited at room temperature on Si(100) substrates via RF sputtering using a pure 2″ diameter Mo target (99.99% purity) in Ar gas atmosphere with a flow rate of 10 sccm. The plasma was obtained by setting the RF power to 100 W, while careful minimizing the reflected power. Before deposition, the target surface was pre-sputtered for 15 min to remove any surface contamination. The silicon substrates were meticulously cleaned by washing them with distilled water and isopropyl alcohol and rinsing them with acetone. The vacuum chamber was evacuated to a base pressure of 2.0 × 10^−3^ mbar, with a working pressure during deposition of approximately 1.2 × 10^−2^ mbar; deposition times varied from 7 to 12 min, resulting in films with thicknesses from 150 to 300 nm, as measured by spectroscopic ellipsometry. After deposition, some films with different thicknesses were implanted with 1 × 10^17^ N_2_^+^·cm^−2^ at 30 keV using a current density of 4 µA·cm^−2^.

The structural properties of the deposited Mo thin films were investigated using a GXRD Bruker AXS GmbH D8 Advance X-ray diffractometer in grazing incidence geometry, employing Cu Kα radiation with a wavelength of 1.5405 Å. Measurements were conducted with a fixed incident angle of 0.5°, and the X-ray tube was operated at 40 kV and 40 mA. The surface morphology was analyzed using a Bruker Multimode-8 atomic force microscopy (AFM). The optical characteristics of the molybdenum thin films were analyzed using a SENresearch 4.0 spectroscopic ellipsometer across a wide spectral range of 200–800 nm. The electrical properties of the films were evaluated using a Keithley 4200 A-SCS parametric analyzer equipped with two probes. All these analytical instruments are available at the Ion Beam Centre, Kurukshetra University, Kurukshetra, ensuring comprehensive and accurate characterization of the properties of Mo thin films.

## Results and Discussion

### SRIM-TRIM simulation

**Figure 1:**
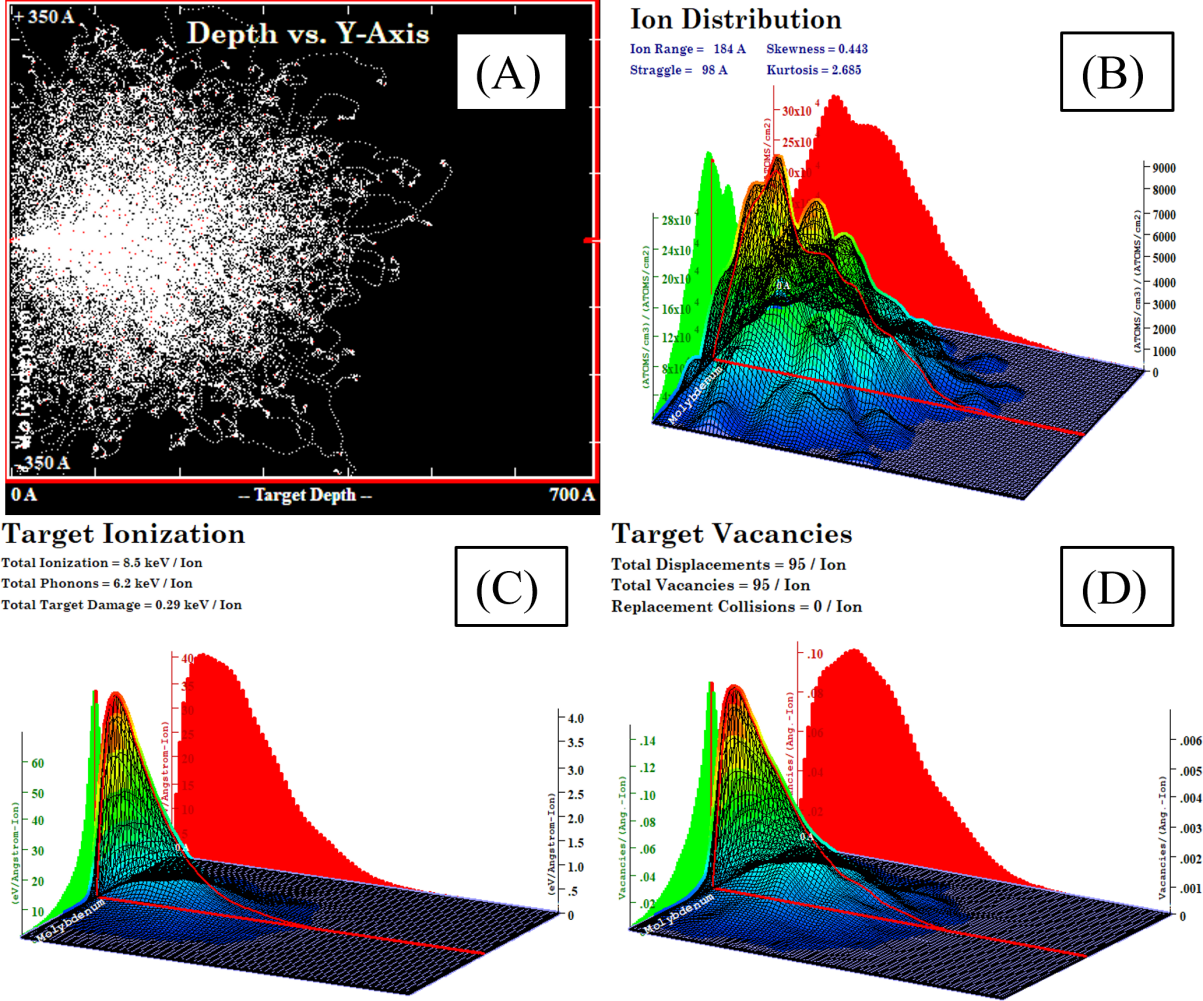
(A) Nitrogen ion trajectories, (B) distribution of ions, (C) distribution of losses due to ionization, and (D) vacancy distribution in a Mo thin film.

The “Stopping & Range of Ions in Matter and Transport of Ions in Matter” (SRIM-TRIM) simulation software was used to quantitatively estimate implantation-induced damages. These calculations provide detailed insights into the distribution of incident ions and the ion damage cascades within the molybdenum target material. The range of the nitrogen ions is 184 ± 98 Å, which is significantly less than the thickness of the thin films, as shown in Figure 1A,B.

The values for electronic energy losses and nuclear energy losses have been determined to be 25.6 and 18.2 eV·Å^−1^, respectively. Both energy loss processes have values within a similar range, which suggests that contributions from both processes are approximately equivalent in their effect on the target material. This balance highlights the importance of electronic and nuclear interactions in the energy transfer processes during ion implantation. Furthermore, of the total energy of 15 keV from a single nitrogen ion, 8.5 keV produce ionization, while 6.2 keV generate phonons, and 0.29 keV create damage within the target material, as demonstrated in Figure 1C.

These findings highlight the significant interactions between the nitrogen ions and the molybdenum target material during the implantation process, providing valuable insights into the mechanisms that govern ion implantation and the subsequent alterations in the material properties. As indicated by the TRIM simulations, a single nitrogen ion with this energy can produce 95 displacements, that is, 95 vacancies and no replacement collisions, before coming to a stop, as illustrated in Figure 1D. This significant level of displacement underscores the ions’ capacity to induce damage within the target material.

### Structural characteristics

**Figure 2:**
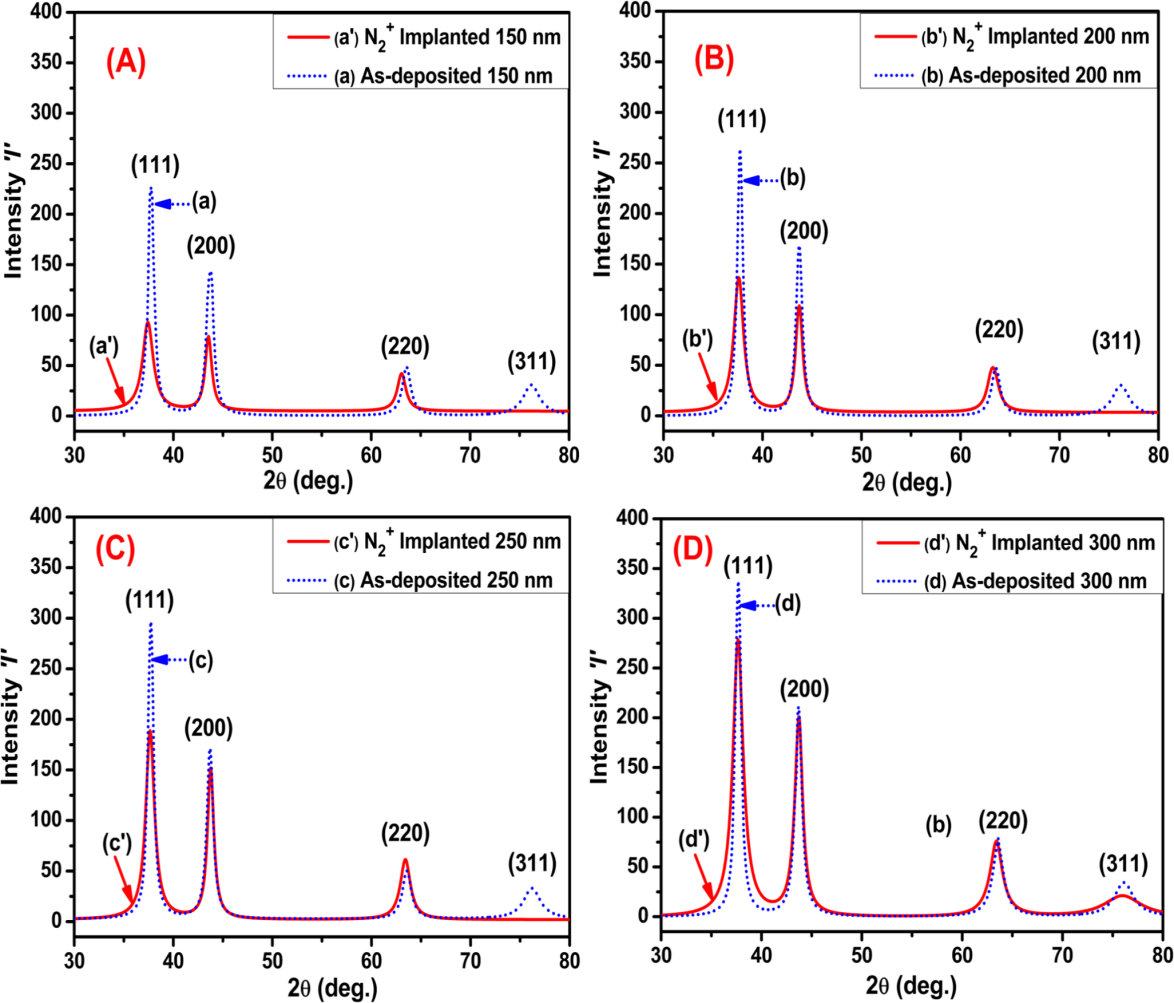
GXRD patterns of as-deposited and N_2_^+^-implanted Mo thin films. (A) 150 nm, (B) 200 nm, (C) 250 nm, and (D) 300 nm.

Figure 2 depicts the GXRD patterns of as-deposited and implanted Mo thin films (at an ion fluence of 1 × 10^17^ N_2_^+^·cm^−2^) with different thicknesses of 150, 200, 250, and 300 nm. The patterns indicate the face-centered cubic Mo phase, as per JCPDS no. 01-088-2331 [29].

In Figure 2A, there is a notable reduction in peak intensity of the Mo film after N_2_^+^ implantation relative to the as-deposited Mo film. This reveals a significant alteration in structure due to the implantation process. Similarly, in Figure 2B–D, the peak intensity of implanted Mo films decreases in comparison to the as-deposited Mo films of the same nominal thickness. This reduced intensity of diffraction peaks is attributed to amorphization and degradation of the structure by N_2_^+^ implantation. However, the peak intensity of the as-deposited Mo films increased with increasing thickness. This trend of intensity with thickness remains in the nitrogen-implanted films. At lower thicknesses (150 and 200 nm), crystallinity is significantly reduced because of the extensive penetration of nitrogen ions, resulting in considerable distortion of the material structure. In contrast, films of 250 and 300 nm maintain a degree of crystallinity derived from the unexposed bulk layer under the ion-implanted layer [30].

The full width at half maximum (FWHM) of diffraction peaks was evaluated from the prominent peak at 2θ = 37.80° associated with the (111) crystallographic plane. Further, the average crystallite size was calculated using the Scherrer equation [31–33]:


[1] $$D=\frac{k\lambda }{\beta \cos\theta ,}$$

where *k* is a constant (*k* = 0.94) [32–33], λ is the X-ray wavelength (λ = 1.5406 Å), θ is the Bragg angle, and β is the FWHM.

Additionally, the microstrain (ε) developed in the thin films due to lattice distortions or mismatch was calculated using Wilson’s equation [31,33]:


[2] $$\varepsilon =\frac{\beta }{4\tan\theta }.$$

The dislocation density (δ) gives more information about the number of defects in the films; it was calculated from the relation [32]:


[3] $$\delta =\frac{1}{D^{2}},$$

where *D* is the average crystallite size.

The interplanar spacing (*d*) and lattice constant (*a*) were calculated using the relations [13,33]:


[4] $$d=\frac{\lambda }{2\sin\theta }\text{ and}$$

[5] $$a=d\sqrt{h^{2}+k^{2}+l^{2}},$$

where *h,k,* and *l* are the Miller indices of the corresponding diffraction peak [34]. The obtained values for average crystallite size, microstrain, dislocation density, and interplanar spacing for as-deposited and N_2_^+^-implanted Mo films are summarized in Table 1.

**Table 1:** Structural parameters of as-deposited and implanted Mo thin films.

Serial number	Sample	2θ (^o^) of the (111) peak	FWHM	Crystallite size *D* (nm)	Microstrain ε (× 10^−3^)	Dislocation density δ (× 10^15^ nm^−2^)	Interatomic spacing *d*(111) (Å)

1	as-deposited 150 nm	37.75	0.603	13.202 ± 1.051	7.542	5.951	2.381
2	N_2_^+^-implanted 150 nm	37.40	0.706	11.921 ± 0.953	9.041	7.032	2.389
3	as-deposited 200 nm	37.73	0.584	14.424 ± 1.131	7.431	4.801	2.382
4	N_2_^+^-implanted 200 nm	37.57	0.685	12.882 ± 1.320	8.745	6.631	2.388
5	as-deposited 250 nm	37.71	0.570	14.981 ± 1.101	7.252	4.472	2.384
6	N_2_^+^-implanted 250 nm	37.60	0.654	13.871 ± 0.789	8.344	5.191	2.387
7	as-deposited 300 nm	37.67	0.552	15.242 ± 1.033	7.041	4.306	2.385
8	N_2_^+^-implanted 300 nm	37.62	0.627	14.424 ± 0.848	7.981	4.802	2.386

Table 1 shows that the FWHM decreases with increase in film thickness. This reveals an enhancement in crystallite size and uniformity, signifying improved crystallinity in the films. However, after N_2_^+^ implantation, the FWHM increases significantly. The crystallite size of the as-deposited and N_2_^+^-implanted Mo films increases from 13.22 to 15.24 nm and from 11.92 to 14.42 nm with an increase in thickness from 150 to 300 nm, respectively. This variation may relate to several factors involving film growth and substrate interactions. Thicker films (i.e., 250 and 300 nm) exhibit less surface diffusion effects during deposition, promoting better crystal formation and atomic plane organization. The increased film thickness may also enable more volume for the formation of ordered crystalline domains [35–36].

After N_2_^+^ implantation, the crystallite size decreased significantly relative to as-deposited Mo films of the same nominal thickness, as illustrated in Table 1. Nitrogen ions during implantation induced many lattice defects, including point defects, dislocations, and distortions, in the Mo crystal lattice, resulting in microstructural disorder and amorphization. The simultaneous impact of strain and defects leads to peak broadening and increase of the FWHM. The disruption of the crystal lattice and an increasing number of defects substantially influenced the grain boundaries, resulting in a notable alteration of the structural parameters of Mo thin films. Additionally, Table 1 shows that microstrain and dislocation density increased after implantation. The interplanar spacing of Mo thin films increased with film thickness [13,34].

**Figure 3:**
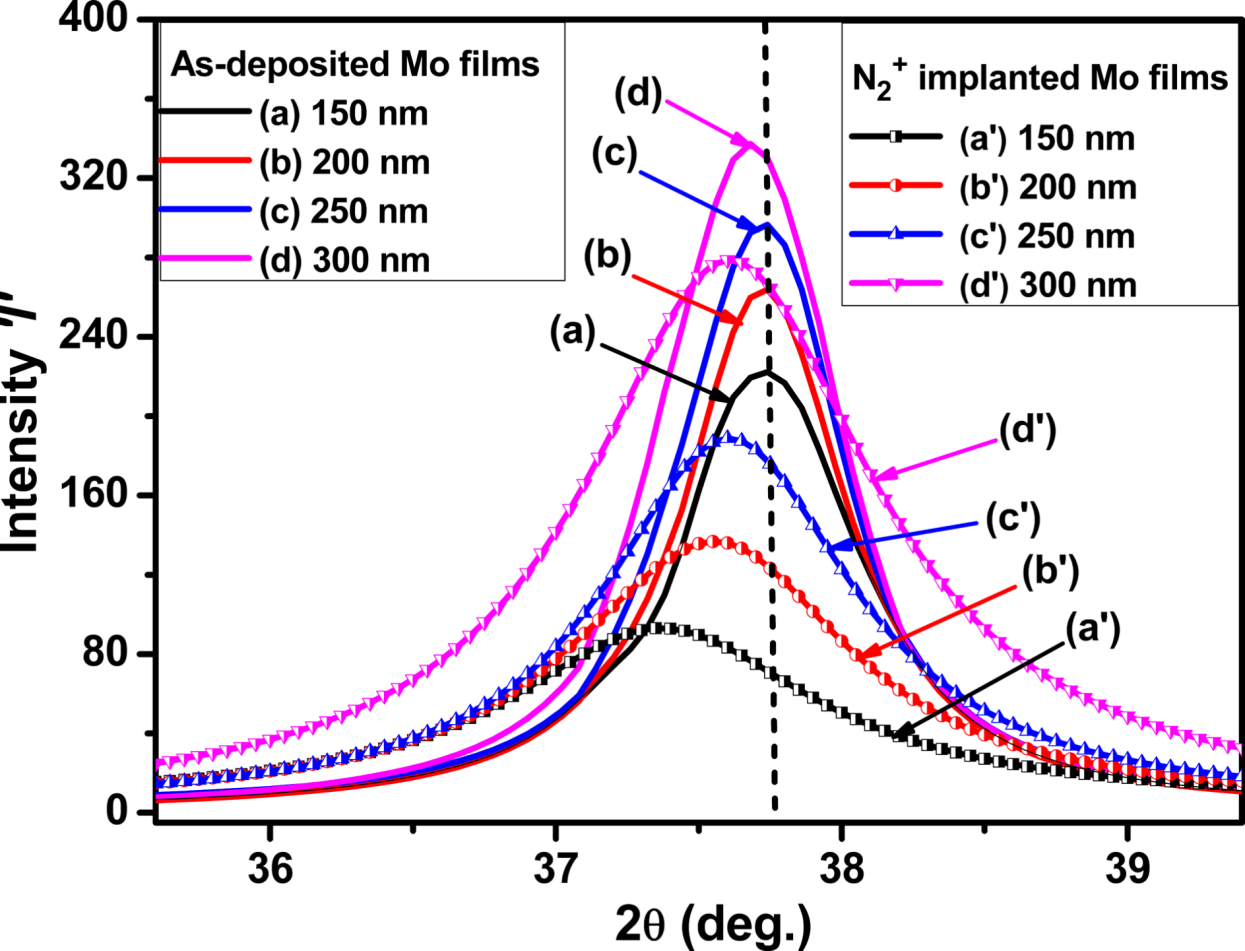
GXRD pattern of the (111) diffraction peak of as-deposited and N_2_^+^-implanted Mo thin films (a, a′) 150 nm, (b, b′) 200 nm, (c, c′) 250 nm, and (d, d′) 300 nm.

Figure 3 shows the GXRD pattern for (111) diffraction peaks of as-deposited and implanted Mo thin films. The (111) peak position shifts slightly to smaller Bragg angles after N_2_^+^ implantation. This shift becomes less pronounced as the thickness increases, indicating an increase in the average interplanar spacing according to Bragg’s law [29,35]. The change in interplanar spacing is attributed to the strain associated with residual stress in the Mo film. The stress induced in the films is calculated using the formula [13,33,37]


**Figure 4:**
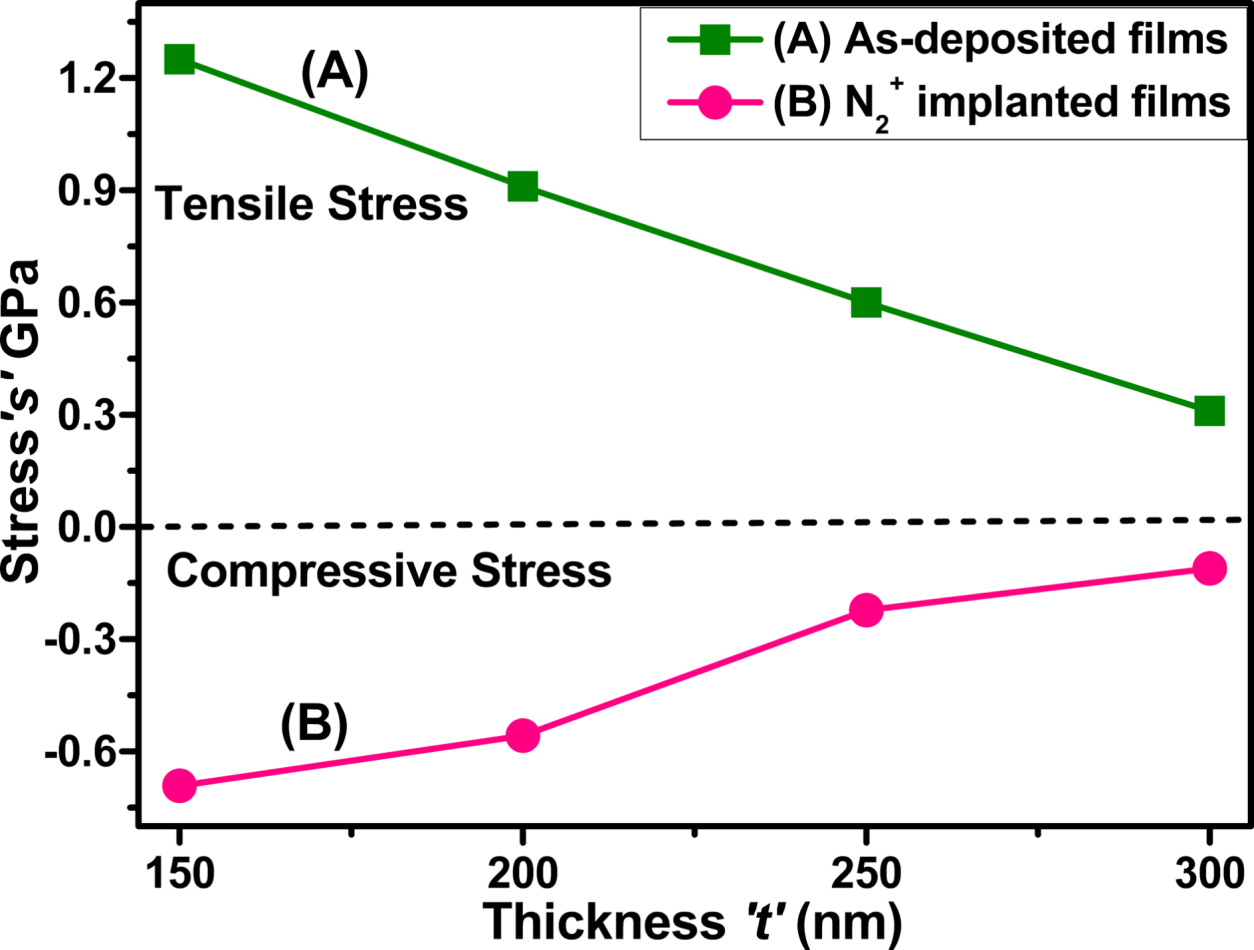
Stress in (A) as-deposited and (B) N_2_^+^-implanted Mo thin films as a function of the thickness.

[6] $$s=\frac{E_{\text{f}}\left(d_{0}-d\right)}{2\nu_{\text{f}}d_{0}},$$

where *E*_f_ is the Young’s modulus, ν_f_ represents the Poisson's ratio, *d*_0_ corresponds to the bulk interplanar spacing, and *d* is the calculated interplanar spacing. The material parameters for Mo are *E*_f_ = 320 GPa, v_f_ = 0.3 [13,33,37], and *d*_0_ = 2.3867 Å [37] (JCPDS Card No. 01-088-2331). The obtained values of stress are plotted in Figure 4.

The as-deposited molybdenum thin films exhibited tensile stress, with the degree of this stress decreasing with an increase in film thickness. After nitrogen ion implantation, the stress in the films changed from tensile to compressive, as illustrated in Figure 4. The X-ray diffraction (XRD) peak position shift provides further insight into these stress changes. In as-deposited films, the peaks are shifting to lower 2θ values, signifying an increase in interatomic separation, which aligns with the measured tensile stress. In the nitrogen-implanted films, all peaks are shifted to reduced 2θ values compared to the as-deposited films, indicating compressive stress. Nevertheless, the peak positions for the implanted films shifted towards increasing 2θ values as the film thickness increased from 150 to 300 nm, signifying a reduction in interplanar spacing. The decrease in interplanar spacing is a direct consequence of the compressive force induced by nitrogen implantation.

During the deposition process, Mo thin films exhibit different types of stress due to changes in microstructure and the interplay of intrinsic and extrinsic stress mechanisms. Figure 4 shows that the tensile stress in as-deposited Mo thin films decreases from 1.25 to 0.31 GPa as the film thickness increases from 150 to 300 nm, attributed to the relaxation of intrinsic stress induced during deposition. At lower thickness, the films exhibit higher tensile stress due to grain boundary contacts and point defects generated during the first phase of film growth. The increased thickness of films led to larger crystallites and improved crystallinity, which reduced tensile stress by decreasing atomic-scale defects and strain [13,29]. The compressive stress increases from −0.69 to −0.11 GPa as the thickness of nitrogen-implanted Mo thin films increases from 150 to 300 nm. Nitrogen ion implantation plays an important role in altering the stress state from tensile to compressive. This process can be explained by the introduction of nitrogen ions into the film structure. This generates compressive stress due to the presence of implanted nitrogen atoms in interstitial positions within the Mo lattice, leading to lattice distortion [38].

As the film thickness increases, nitrogen ions are dispersed over more volume, enhancing the cumulative distortion effects inside the films. Furthermore, nitrogen implantation improves densification and modifies the crystal structure. Nitrogen ions serve as a barrier in boundary relaxation and grain growth, hence increasing the stress. The divergent behavior illustrates the significant influence of nitrogen inclusion on the stress evolution of Mo thin films.

The crystalline orientation of the Mo films along a particular (*hkl*) plane can be quantified by determining the texture coefficient (TC*_hkl_*), which is determined by the following equation [39–40]:


[7] $${\text{TC}}_{hkl}=\frac{I\left(hkl\right)}{I\left(111\right)+I\left(200\right)+I\left(220\right)+I\left(311\right)},$$

where *hkl* represents the (111), (200), (220), or (311) orientations. The calculated TC*_hkl_* values for each Mo thin film are summarized in Table 2.

**Table 2:** Lattice constants and texture coefficients of as-deposited and implanted Mo thin films.

Serial number	Sample	Lattice constant *a* (Å)	Texture coefficient			

			TC_(111)_	TC_(200)_	TC_(220)_	TC_(311)_

1	as-deposited 150 nm	4.124	0.501	0.302	0.104	0.054
2	N_2_^+^-implanted 150 nm	4.139	0.433	0.364	0.172	0.000
3	as-deposited 200 nm	4.126	0.511	0.311	0.110	0.057
4	N_2_^+^-implanted 200 nm	4.138	0.462	0.373	0.183	0.000
5	as-deposited 250 nm	4.129	0.518	0.328	0.118	0.059
6	N_2_^+^-implanted 250 nm	4.135	0.482	0.384	0.189	0.000
7	as-deposited 300 nm	4.133	0.525	0.331	0.126	0.061
8	N_2_^+^-implanted 300 nm	4.134	0.492	0.391	0.191	0.035

The texture coefficients of as-deposited and N_2_^+^-implanted Mo thin films demonstrate a strong orientation preference in the (111) direction. The TC_111_ values of these films are significantly larger than those for other orientations. Also, the texture coefficient for the (200) plane, though less than that for the (111) plane, is larger than those for the (220) and (311) planes. These observations reveal that the plane (111) is the dominant crystalline orientation in both as-deposited and nitrogen-implanted Mo thin films. The (111) plane in face-centered cubic structures, such as Mo, typically has the lowest surface energy [35]. Therefore, atoms tend to arrange themselves to minimize the overall energy, leading to a predominant (111) orientation. This tendency is even more pronounced for as-deposited films, where the absence of any external influence would have allowed for the natural minimization of surface energy. Nitrogen ion implantation could change the crystalline orientation through defects and stress introduced in the Mo thin films. The residual stress due to the implantation may also be an influential factor regarding the preferred orientation. If the applied stress promotes the (111) orientation, this plane will be more pronounced in the resultant thin film [9].

Additionally, the texture coefficients increase with the thickness for both as-deposited and implanted Mo thin films. Thicker films (i.e., 250 and 300 nm) generally exhibit stronger orientation preferences because of the increased volume for developing certain planes. In contrast, thinner films (i.e., 150 and 200 nm) exhibit a specific orientation preference owing to the restricted volume, which restricts the formation of certain planes [41]. Furthermore, the interaction between the substrate and the thin film can generate stress and strain, affecting the growth of specific planes. If the substrate promotes the (111) orientation through lattice compatibility, the resultant Mo thin film exhibits a pronounced (111) orientation.

Crystallite size and texture coefficients of these Mo thin films significantly increase with the thickness of Mo thin films. Moreover, a significant reduction has been observed in both these parameters after nitrogen implantation compared to equivalently thick as-deposited films. The increase in crystallite size with greater film thickness directly signifies enhanced crystallinity, observable by the formation of larger crystallites in thicker films. Additionally, the texture coefficient rises, signifying that crystallites attain a more uniform orientation in the film.

### Surface morphology

**Figure 5:**
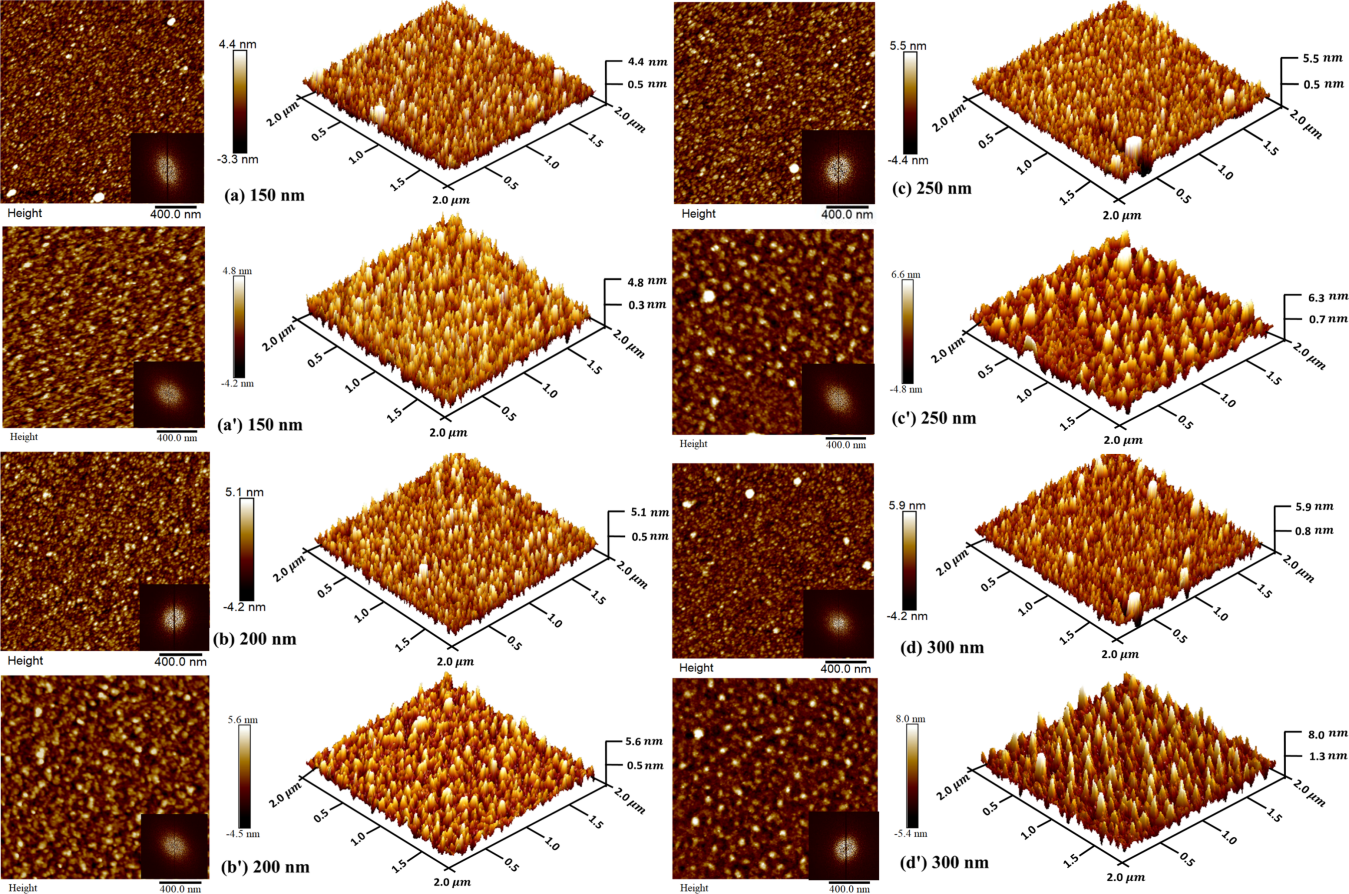
2D and 3D AFM images of Mo thin films: as-deposited (a) 150 nm, (b) 200 nm, (c) 250 nm, and (d) 300 nm, and implanted (a′) 150 nm, (b′) 200 nm, (c′) 250 nm, and (d′) 300 nm. The fast Fourier transform images are insets in the 2D AFM micrographs.

Figure 5 depicts 2 × 2 µm^2^ 2D and 3D AFM images of as-deposited and implanted (at an ion fluence of 1 × 10^17^ N_2_^+^·cm^−2^) Mo thin films. Figure 5a reveals that the 150 nm thick Mo film exhibits a surface with thinner and more elongated rough peaks [42]. In this stage of deposition, the particles exhibit granular, tightly packed vertical peak growth. After nitrogen implantation (Figure 5a′), the shape altered into broader and more flatter characteristics [42]. This may be because of surface alterations induced by the N_2_^+^ implantation [9,42]. Interestingly, variations in Mo film thickness from 150 to 300 nm also led to noticeable changes in morphology. With the increase in thickness, rough peaks evolved into broader and flatter shapes. The fast Fourier transform images indicate random distribution with different orientations of particles. The surface morphology parameters are listed in Table 3. The RMS roughness values of Mo thin films with 150, 200, 250, and 300 nm thickness are around 1.09, 1.23, 1.41, and 1.73 nm, respectively. After N_2_^+^ implantation, the RMS roughness increases to 1.28, 1.43, 1.58, and 1.89 nm, respectively. A similar trend in roughness has been observed after N_2_^+^ implantation for all Mo films. Table 3 reveals a continuous increase in particle size with Mo film thickness before and after N_2_^+^ implantation.

**Table 3:** Values of RMS surface roughness and particle size of as-deposited and implanted Mo thin films.

Serial number	Sample	Roughness (nm)	Particle size (nm)

1	as-deposited 150 nm	1.09 ± 0.11	67.41 ± 0.88
2	N_2_^+^-implanted 150 nm	1.28 ± 0.21	100.71 ± 1.23
3	as-deposited 200 nm	1.23 ± 0.32	73.26 ± 1.21
4	N_2_^+^-implanted 200 nm	1.43 ± 0.12	106.41 ± 1.11
5	as-deposited 250 nm	1.41 ± 0.14	80.86 ± 1.24
6	N_2_^+^-implanted 250 nm	1.58 ± 0.28	113.24 ± 1.31
7	as-deposited 300 nm	1.73 ± 0.16	88.24 ± 1.40
8	N_2_^+-^implanted 300 nm	1.89 ± 0.21	119.86 ± 1.43

This increase in roughness and particle size is related to structural alterations due to the increasing film thickness. At the lowest thickness, a greater number of particles are found to be small [43]. As the thickness of Mo thin films increases, smaller particles coalesce, which increases the surface roughness. The increase in roughness and particle size of implanted films results from structural modifications caused by N_2_^+^ implantation. AFM analysis reveals that N_2_^+^ implantation and thickness of films significantly influence the surface morphology of Mo thin films.

### Optical properties

#### Spectroscopic ellipsometry

**Figure 6:**
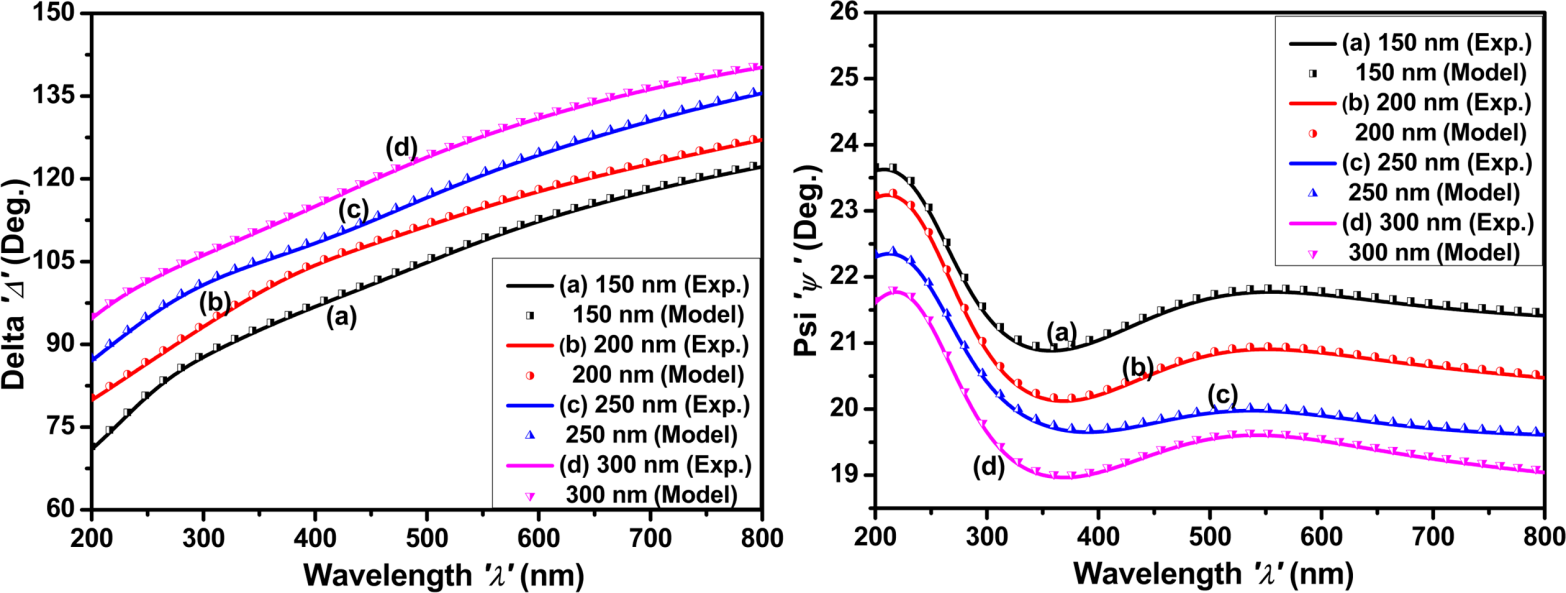
Measured SE data (solid line) and fit (symbols) of ψ and Δ spectra as functions of the wavelength of N_2_^+^-implanted molybdenum thin films with thicknesses of (a) 150 nm, (b) 200 nm, (c) 250 nm, and (d) 300 nm.

Figure 6 depicts the fit of the spectroscopic ellipsometry data in which the parameters of the Drude–Lorentz oscillator model were adjusted. In ellipsometry, the two parameters ψ and Δ characterize a change in polarization state. ψ denotes the amplitude ratio between p-polarized (parallel to the plane of incidence) and s-polarized (perpendicular to the plane of incidence) light components after interaction with the sample. Δ denotes the phase difference between these components after contact with the sample. Upon successfully fitting the results of N_2_^+^-implanted molybdenum thin films for all thickness, a small error percentage (in this case, 0.15%) between the experimental and simulated curves is observed, indicating a close match [2,44]. The simulated curves derived from the fit process validate the results and offer a theoretical framework for understanding the optical properties of molybdenum thin films.

#### Absorbance spectra

**Figure 7:**
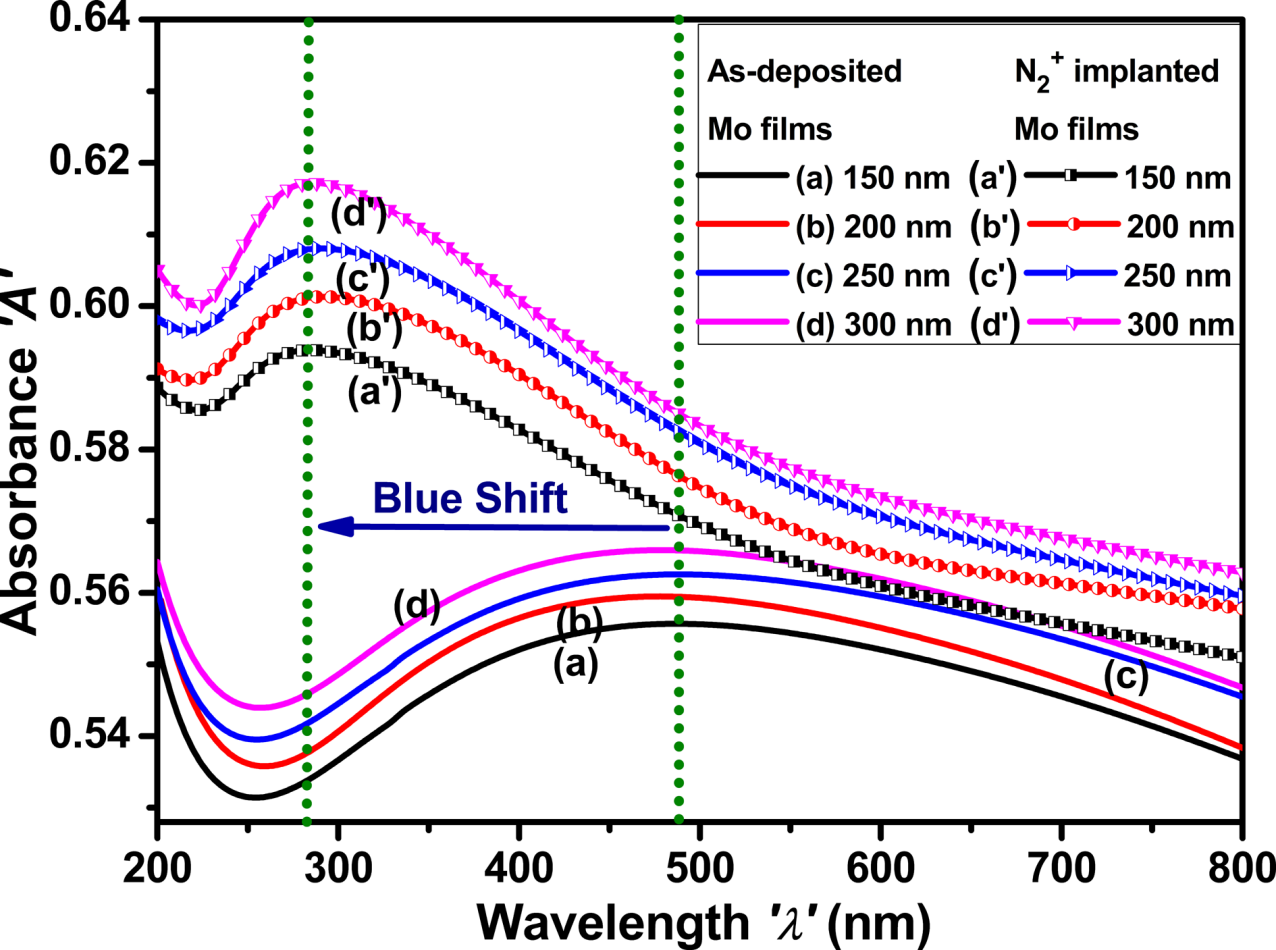
Absorbance spectra of as-deposited and N_2_^+^-implanted Mo thin films with thicknesses of (a, a′) 150 nm, (b, b′) 200 nm, (c, c′) 250 nm, and (d, d′) 300 nm.

The absorption spectra of Mo thin films in Figure 7 show the effects of nitrogen ion implantation and film thickness on the production of Mo nanoparticles and the subsequent increase in surface plasmon resonance or interband transitions. As-deposited molybdenum thin films deposited at room temperature typically exhibit a smooth surface and crystalline structure. The absorbance of molybdenum thin films increases with increasing film thickness and peaks between 300 and 700 nm, corresponding to the localized surface plasmon resonance (LSPR) of molybdenum nanoparticles [45–46].

The formation of defects through ion irradiation increased the scattering of light and absorption within the film, resulting in enhanced overall absorbance of Mo thin films. The blue shift of the LSPR band to a shorter wavelength (i.e., 260–340 nm) after nitrogen ion implantation can be attributed to modifications in the size, shape, and density of molybdenum nanoparticles [47]. The absorbance bands are more pronounced after nitrogen implantation because of defects that facilitate the growth of smaller nanoparticles or cause aggregation, consequently altering the plasmonic characteristics of the material.

#### Reflectance spectra

**Figure 8:**
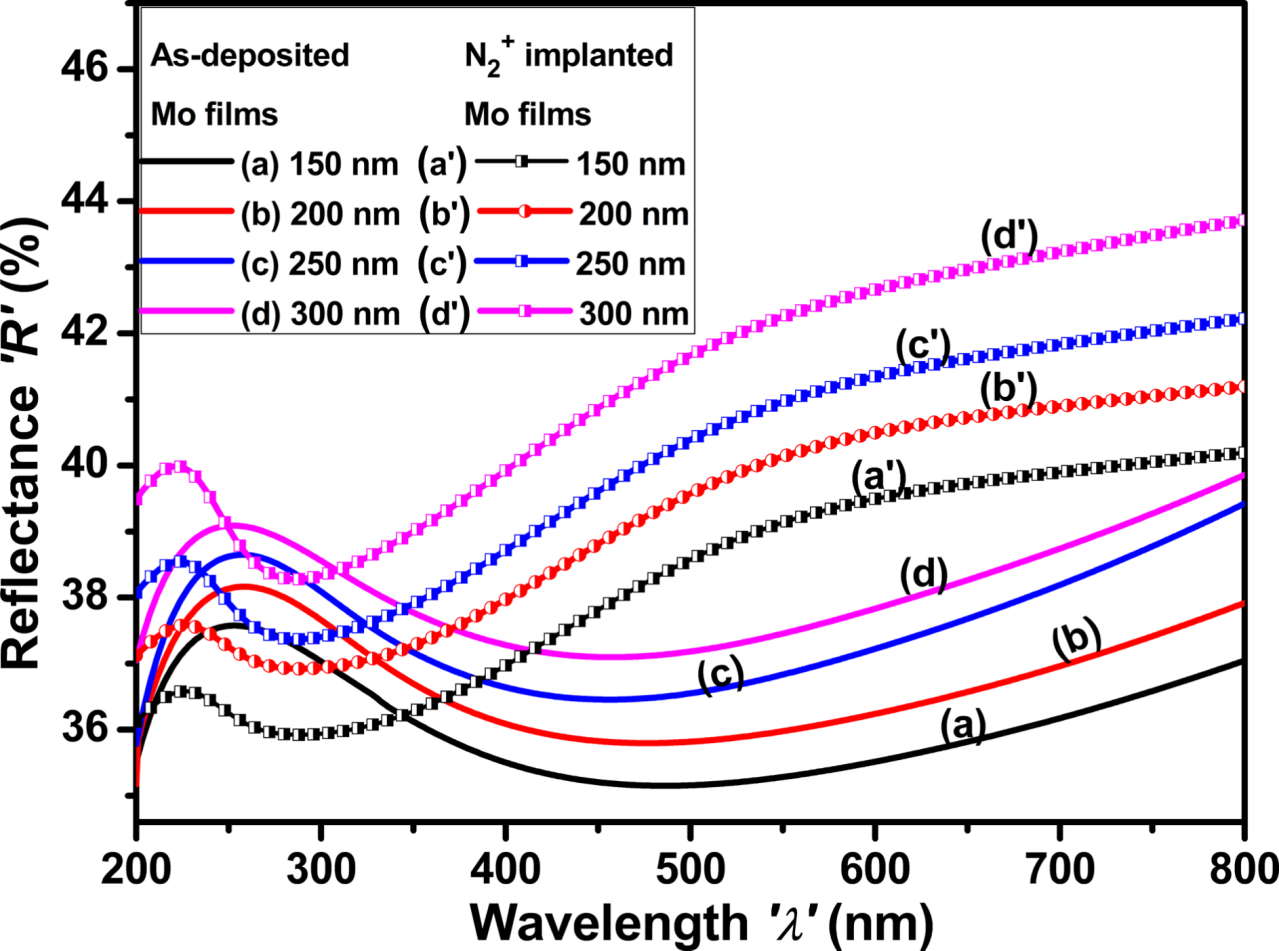
Reflectance spectra of as-deposited and N_2_^+^-implanted Mo thin films with thicknesses of (a, a′) 150 nm, (b, b′) 200 nm, (c, c′) 250 nm, and (d, d′) 300 nm.

Figure 8 shows the reflectance spectra as-deposited and implanted Mo thin films as functions of the thickness. The dip observed in the reflectance spectra of Mo films between 300 and 700 nm is attributed to the LSPR of Mo nanoparticles. The peak observed in the reflectance spectra at approximately 260 nm correlates with the dip in absorbance [48]. A notable enhancement in the reflectance of Mo thin films is observed as a function of the film thickness. Ion implantation induces many alterations in the reflectance spectra. The shift of the peak to shorter wavelengths (about 225 nm) shows a change in the electrical structure of the Mo thin film, probably due to defect formation. These defects can modify the plasmonic behavior of the nanoparticles, influencing their resonance properties and, subsequently, the absorbance spectrum [49]. Furthermore, the alteration in peak location indicates an increased number of nanoparticles or a modification in their size distribution resulting from damage caused by ion implantation.

#### Refractive index

**Figure 9:**
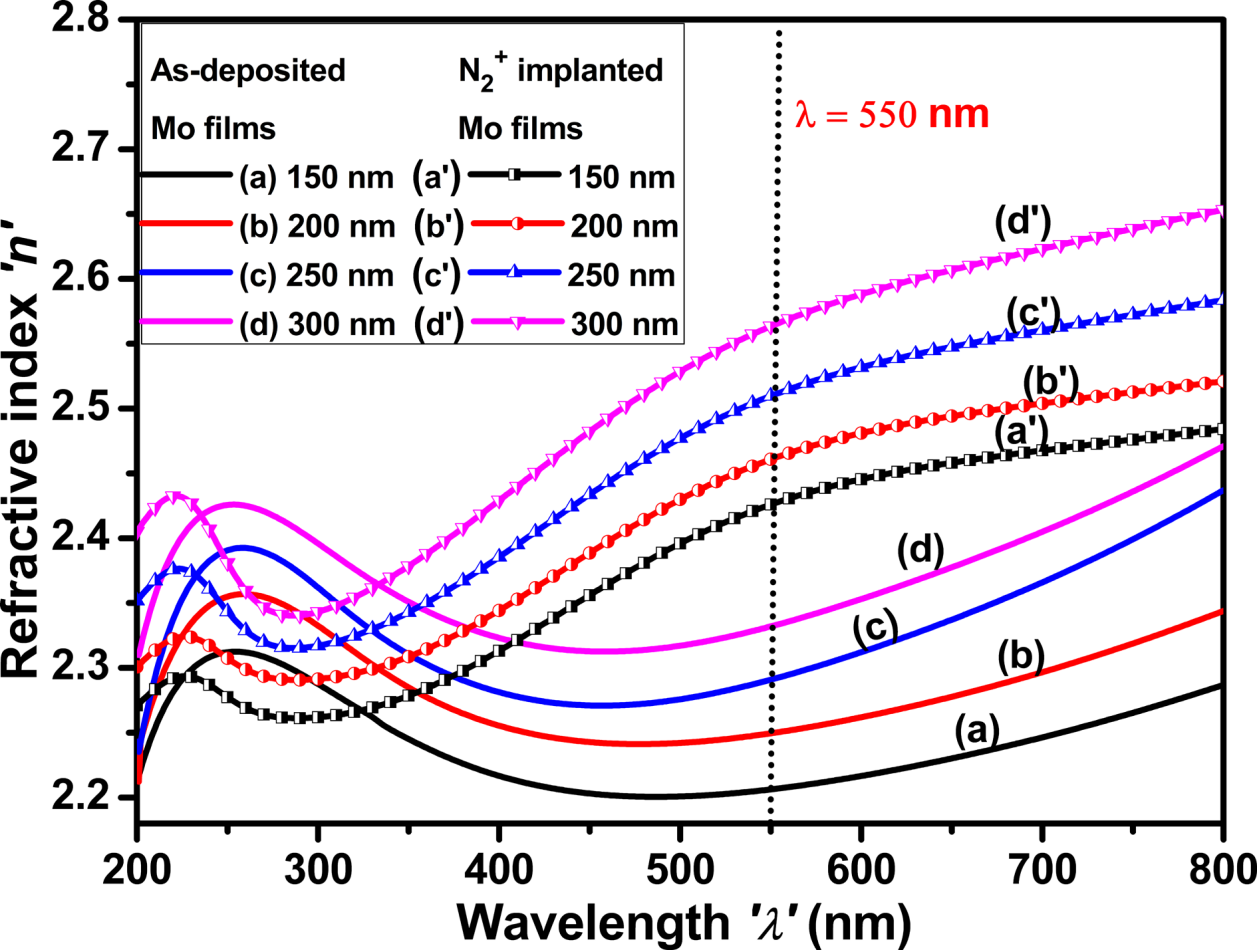
Refractive index of as-deposited and N_2_^+^-implanted Mo thin films with thicknesses of (a, a′) 150 nm, (b, b′) 200 nm, (c, c′) 250 nm, and (d, d′) 300 nm.

Figure 9 shows the refractive index of as-deposited and implanted Mo thin films as a function of the thickness. The refractive index increases after N_2_^+^ implantation because of several factors, notably the formation of defects and changes in the composition and structure of Mo thin films. There is a dip in the refractive index between 300 and 700 nm, attributed to the LSPR of molybdenum nanoparticles. Defects include interstitials, vacancies, and structural deformation, which modify the electrical and lattice structure of the material [49–50]. The refractive index of both as-deposited and implanted Mo thin films increases with increasing film thickness. The increased refractive index suggests increased particle density and a better interaction between light and Mo thin films.

### Electrical properties

**Figure 10:**
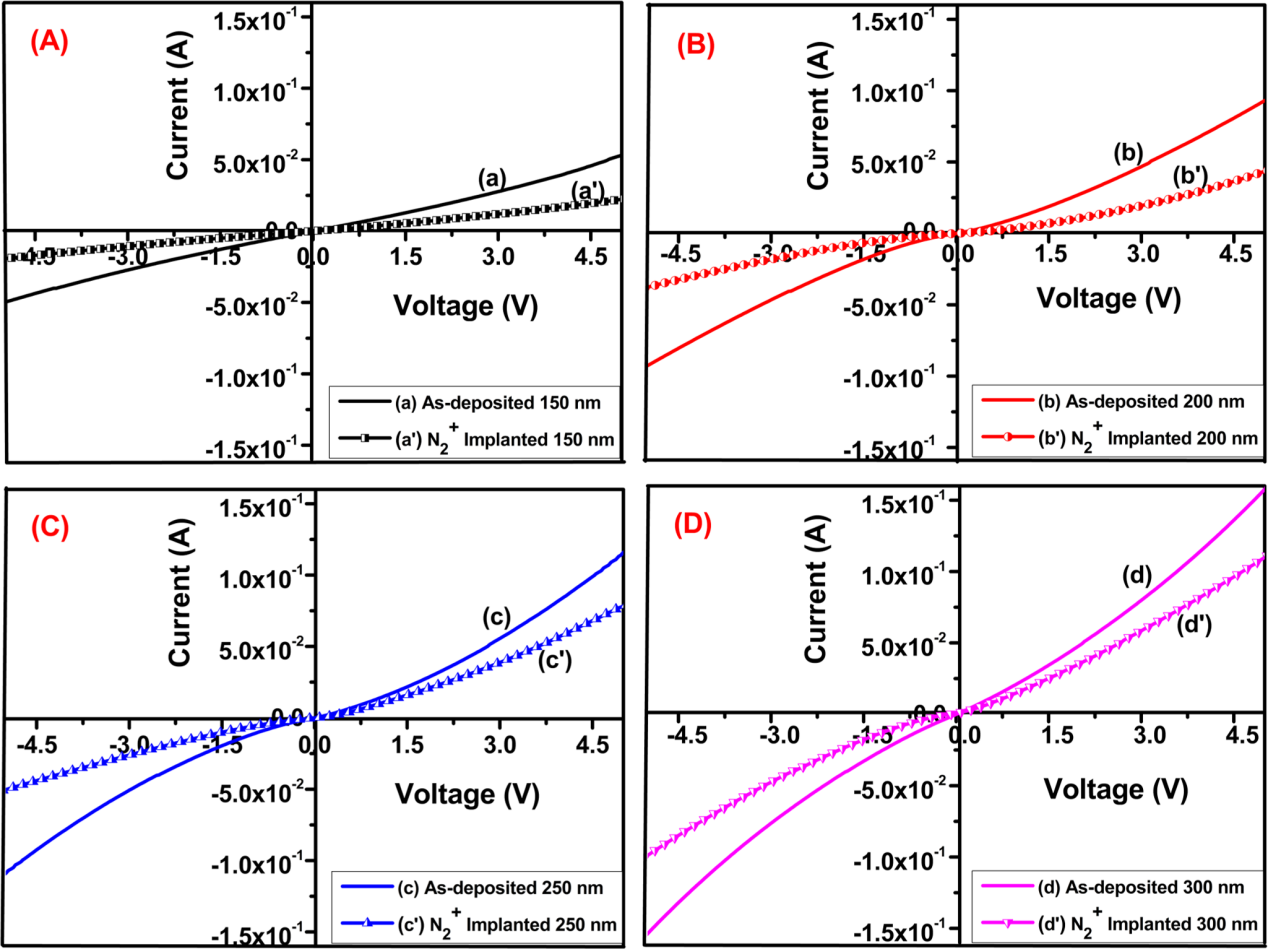
Current–voltage characteristics of as-deposited and N_2_^+^-implanted Mo thin films with thicknesses of (A) 150 nm, (B) 200 nm, (C) 250 nm, and (D) 300 nm.

Figure 10 shows the current–voltage (*I*–*V*) characteristics of the as-deposited and implanted Mo thin films as a function of thickness. *I*–*V* curves have been obtained within a voltage range of −5 to 5 V. A nearly linear relationship between applied voltage and the resulting current indicates ohmic behavior [51]. Resistance and resistivity of the Mo thin films have been calculated based on the slope of the *I*–*V* curve. The observed increase in current across the thin films suggests their superior electrical conductivity. Such characteristics are highly desirable for various electronic and electrical applications where efficient electrical conduction is paramount.

The stress in the films may influence the electrical properties. As the thickness changes, the internal stress distribution could lead to impediments in electron transport, causing slight deviations from linearity. Nitrogen ions create defects, vacancies, or impurities in the molybdenum film, acting as scattering centers for charge carriers. Ion implantation may also create localized states within the film, causing some charge carriers to become trapped. This affects the charge transport mechanism, leading to slight nonlinearities. The ion implantation causes modifications in the grain structure of the molybdenum thin films. Such structural changes can influence the movement of electrons through the material, particularly at grain boundaries, resulting in nonlinear *I*–*V* behavior.

#### Resistivity and conductivity

The resistivity of thin films is defined by the relation [51–52]:


[8] $$\rho =R\frac{A}{l},$$

where ρ is the resistivity, *R* is the resistance, *A* represents the area of the thin molybdenum film samples (width of film × thickness of film), and *l* corresponds to the length between the two probes. The relationship between resistivity and conductivity is [51]:


[9] $$\sigma =\frac{1}{\rho },$$

where σ represents the conductivity of thin films, and ρ corresponds to the resistivity of thin molybdenum films. The resistivity of the films increases after implantation but reduces as the thickness of the film increases from 150 to 300 nm, as summarized in Table 4.

**Table 4:** The electrical parameters of as-deposited and implanted Mo thin films.

Serial number	Thickness ‘*t*’ (nm)	Resistance ‘*R*’ (Ω)	Resistivity ρ (× 10^−3^ Ω·cm)	Conductivity σ (× 10^2^ S·cm^−1^)

1	as-deposited 150 nm	101	1.51	6.62
2	N_2_^+^-implanted 150 nm	160	2.56	3.90
3	as-deposited 200 nm	72	1.44	6.94
4	N_2_^+^-implanted 200 nm	117	2.40	4.18
5	as-deposited 250 nm	55	1.37	7.27
6	N_2_^+^-implanted 250 nm	90	2.25	4.44
7	as-deposited 300 nm	45	1.32	7.57
8	N_2_^+^-implanted 300 nm	68	2.07	4.84

**Figure 11:**
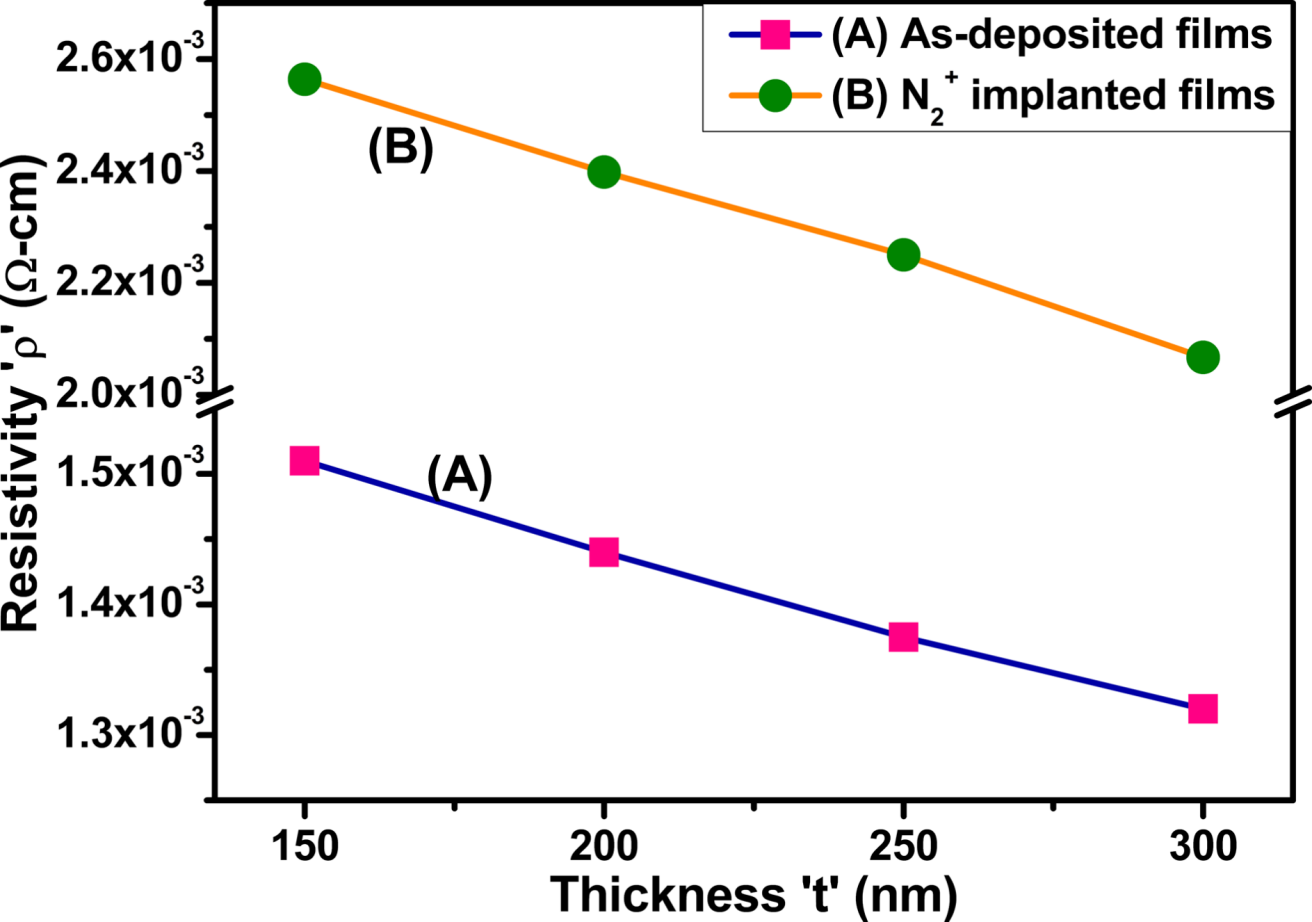
Resistivity of (A) as-deposited and (B) N_2_^+^-implanted Mo thin films as a function of the thickness.

The conductivity of as-deposited Mo thin films increases from 6.62 × 10^2^ to 7.57 × 10^2^ S·cm^−1^ when the film thickness increases from 150 to 300 nm. This can be ascribed to the increased conducting pathways in thicker films. These channels provide more pathways for electrons to flow, enhancing conductivity. Conversely, the conductivity of the thin molybdenum films tends to decrease after N_2_^+^ implantation for films with the same nominal thickness [32,53]. The resistivity of as-deposited and implanted molybdenum thin films with different thickness is illustrated in Figure 11.

The increase in resistivity after nitrogen implantation is linked to the decreased crystalline quality of molybdenum thin films. After implantation, various grain boundaries disrupt the crystal structure. These boundaries act as obstacles for charge carriers, hindering their movement and thus increasing resistivity. Further, scattering at these boundaries reduces the mobility of charge carriers, leading to a decrease in conductivity. Essentially, the more boundaries present after implantation, the greater the scattering effect [32]. As the thickness of the films increases, their conductivity increases. This is because thicker films have a denser microstructure. More atoms are packed closely in thicker films, creating a more efficient pathway for electrical current to travel [54]. The increase in conductivity with thickness is beneficial for applications such as interconnects and conductive traces in microelectronic devices, where efficient electrical conduction is crucial. Overall, ion implantation with a nitrogen ion beam under the specified conditions affects the conductivity of molybdenum thin films by introducing defects, modifying the crystal structure, doping the material with nitrogen ions, and potentially improving surface characteristics.

### Correlation of structural, optical, and electrical parameters with thickness

**Figure 12:**
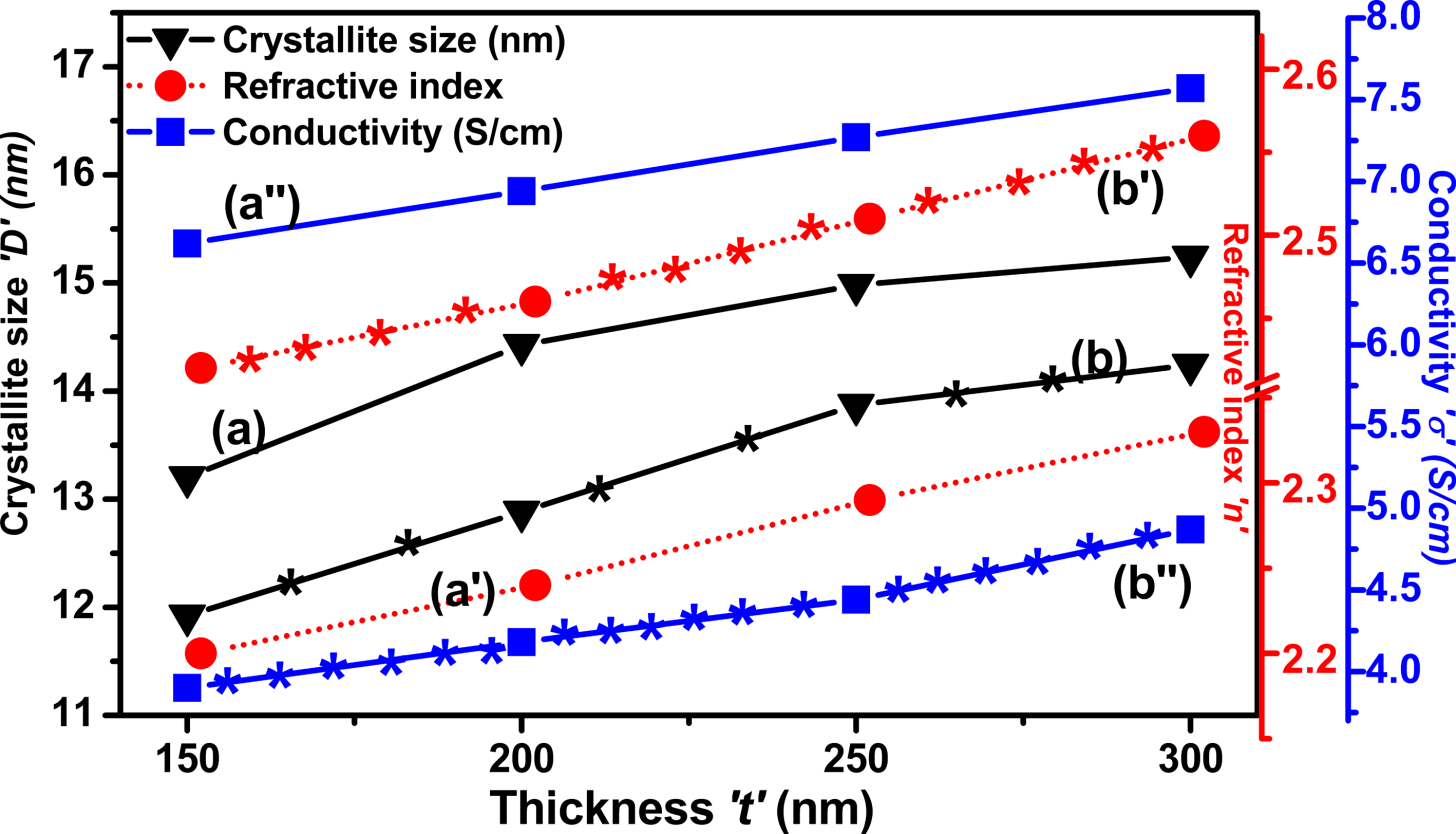
Mutual correlation between (a, b) crystallite size, (a′, b′) refractive index, and (a″, b″) conductivity and thickness of the Mo films. (a), (a′) and (a″) stand for as-deposited and (b), (b)′, and (b″) stand for N_2_^+^-implanted Mo thin films.

Figure 12 depicts the mutual correlations between the structural, optical, and electrical parameters of both as-deposited and N_2_^+^-implanted Mo films as a function of film thickness. The crystallite size of the as-deposited Mo films increased with increasing thickness, signifying a more defined crystalline structure. This trend persists even after ion implantation, with a reduction in crystallite size due to structural alterations and defects introduced by the ion implantation process. After N_2_^+^ implantation, the refractive index increased due to defects created by the implantation, which modified the crystal structure and resulted in a denser, optically distinct material. Similarly, the electrical conductivity of the as-deposited Mo films increased with increasing thickness, as thicker films often provide better pathways for electron conduction because of less scattering effects. However, after implantation, several grain boundaries are compromising the crystalline structure. These barriers act as obstacles for charge carriers, obstructing their mobility and increasing resistivity. This results in a reduction in conductivity. Despite this reduction, the conductivity also increased in implanted films.

## Conclusion

This study gives comprehensive insights into the structural, morphological, optical, and electrical properties of Mo thin films of varying thickness deposited on Si(100) substrates by RF sputtering. Some of these films have been implanted with 1 × 10^17^ N_2_^+^·cm^−2^ at 30 keV. GXRD showed that crystallite size and texture coefficients of Mo thin films enhanced with thickness in as-deposited films, indicating improved crystallographic order and diminishing dislocations and microstrain. This trend with film thickness persists even after N_2_^+^ implantation. After implantation, these parameters significantly decrease relative to as-deposited films with the same nominal thickness. Structural analysis also shows a shift of peaks towards lower 2θ values, indicating that the tensile stress in as-deposited Mo thin films changes to compressive stress after implantation. The AFM analysis shows that RMS roughness increases with thickness in both as-deposited and implanted films. The RMS roughness significantly increases upon implantation. Optical studies using SE show a significant increase in absorbance and reflectance in both as-deposited and N_2_^+^-implanted films. The absorbance peak and reflectance spectra dip correspond to molybdenum nanoparticles. Electrical investigations showed that conductivity increases with the film thickness in both as-deposited and implanted films, but decreases upon implantation for the corresponding films with the same nominal thickness. The findings suggest that nitrogen ion implantation can tailor the properties of Mo thin films, enhancing their performance in applications such as photovoltaic devices, energy storage, and integrated circuits.

## Data Availability

Data generated and analyzed during this study is available from the corresponding author upon reasonable request.
